# Privatized employment services in Australia: addressing social, health, and equity impacts for health promotion

**DOI:** 10.1093/heapro/daag005

**Published:** 2026-02-02

**Authors:** Julia Anaf, Frances Baum, Toby Freeman

**Affiliations:** Stretton Health Equity, School of Public Health, College of Health, Adelaide University, North Tce Adelaide, SA 5005, Australia; Stretton Health Equity, School of Public Health, College of Health, Adelaide University, North Tce Adelaide, SA 5005, Australia; Stretton Health Equity, School of Public Health, College of Health, Adelaide University, North Tce Adelaide, SA 5005, Australia

**Keywords:** privatization, outsourcing, neoliberalism, health, equity, health promotion

## Abstract

Employment status is an important social determinant of health. Employment services mediate unemployed people’s outcomes and experiences, including their psychosocial and physical health and well-being, but have received little health promotion research attention. Over the past 30 years in Australia, employment service provision has been operating under a privatized model with five different policy iterations, yet there has been little consideration of the health impacts of this policy. Documentary methods examined these impacts, producing five key themes: (i) ideological underpinnings of privatization, (ii) the primacy of private interests, (iii) impacts on quality of service, (iv) negative social and health impacts, and (v) implications for equity. Perverse incentives, system gaming, and punitive forms of conditional welfare all lead to negative outcomes, including poverty and severe emotional distress, which unfairly affect people living in disadvantaged circumstances. There are growing calls for much greater direct government involvement in employment services to promote health and equity and public over private interests.

Contribution to Health Promotion

- This study examines the social, health, and equity impacts of privatized employment services from a health promotion perspective, revealing that privatized employment services impact the social determinants of health, are detrimental for well-being, and contribute to inequities.
- It highlights the global ideological underpinnings and Australian historical context of privatized employment services that contribute to unemployment having a negative impact on health.
- There is a strong imperative for the Australian Government to undertake a more directive role with privatized employment services or to bring these back into public hands to promote health and equity.

## Introduction

Health is a positive concept emphasizing social and personal resources, as well as physical capacities. Health promotion is therefore not just the responsibility of the health sector but goes beyond healthy lifestyles to support well-being (World Health Organisation 1986). One critical factor for achieving well-being is employment, with employment status an important social determinant of health (World Health Organisation 2008). Employment is a key condition under which people live and work that influences their chances to lead healthy lives; with health being both differentially and unfairly distributed (World Health Organisation 2008). Promoting sustained, full, and productive employment and decent work for all is also one of the United Nation’s 17 Sustainable Development Goals (United Nations n.d.). The International Labour Organisation (1999, preface) argues that decent work is the ‘most widespread need, shared by people, families and communities in every society, and at all levels of development’ and so is vital to health promotion.

Adequate and efficient employment services are therefore critical for mediating unemployed peoples’ outcomes and experiences, including their psychosocial and physical health and well-being (Australian Institute for Health and Welfare 2023). Yet the impacts of employment services on unemployed people have received little public health research attention. Australia provides an insightful case study of these services which moved from being public to private and have undergone frequent restructuring. Privatized service agencies are profit-seeking entities and thus act as commercial determinants of health, or ‘the systems, practices, and pathways through which commercial actors drive health and equity’ (Gilmore *et al*. 2023, p. 1195).

### Historical background

In 1946, following World War II, as part of the Australian Labor Government’s commitment to full employment, the Commonwealth Employment Service (CES) was established to assist unemployed people to find work. Over 52 years of operation, it also assisted existing workers to pursue different or better jobs (National Employment Services Association n.d.). However, a coercive element, the ‘work test’, required welfare recipients to also prove their ability and willingness to work (Coonan 2020) under a reciprocal obligation with government.

This strong state tradition of Australian Government service provision was challenged in the 1980s when privatization became a widespread approach globally (Aulich and O’Flynn 2007, Anaf *et al*. 2024). Partnerships between public and private organizations were also deemed an important way for governments to develop innovative and cost-effective approaches to delivering public services globally (Hodge and Greve 2007). However, early claims that private financing of public infrastructure reduces pressure on public sector budgets and provides more infrastructure than is otherwise achievable has been seen to be largely false (Hodge and Greve 2007). Australian policies mirrored similar developments in other countries where New Public Management (NPM) techniques were imposed to counter perceptions of public sector inefficiency, purported government failure, and complacency due to a lack of competition (Heins and Bennett 2016).

Part of this rationale was the proclaimed value of flexible private sector financial management (Heins and Bennett 2016). For example, in the USA, the Job Training Partnership Act was established in 1983, including certain minimum performance-based standards, while France abolished the government monopoly on job placement services provision in 2005 (Langenbucher and Vodopivec n.d.). The Australian experience began with privatization of major state-owned assets under the Hawke-Keating Labor Government, with the CES partly privatized in 1994, then fully privatized in 1998 under a Liberal (conservative) Government (O'Sullivan *et al*. 2021b). It adopted a fully contracted-out employment services model, Job Network (National Employment Services Association n.d., O'Sullivan *et al*. 2021b).

Privatization of employment services in Australia also coincided with international policy moves towards conditional welfare, including a debate over ‘welfare dependency’ in the USA under President Reagan and the UK under Prime Minister Thatcher. Underpinning this debate was the view that welfare provision results in an ‘underclass’ of unproductive people. This ideological standpoint influenced Australian views of income-support policy (O'Connor 2001) with support payments considered welfare, thus framing the policy problem as one of welfare dependency rather than as unemployment or poverty (Bacchi 2009, Arthur 2021).

Since the 1990s, the different privatized policy iterations include ‘Working Nation’, ‘Job Network’, ‘Job Services Australia’, ‘Jobactive’, ‘Workforce Australia’, and the introduction of a range of specialized services including the Community Development Program for Aboriginal and Torres Strait Islander people, Disability Employment Services, and Parents Next for parents of young children (National Employment Services Association n.d.). Contracting out employment services in Australia has thus been dubbed ‘the reform that never ends’ (Considine 2005).

As Australia’s employment services have been reviewed with each change of government since 1994 (Table 2), rather than brought back under government control, there is an apparent political consensus that employment services are best delivered by the private sector. The claim that contractualism was the best option for provision of employment services is not unique to Australia but has also endured over different UK governments with increasing escalation of marketized employment services (Heins and Bennett 2016). Extensive literature exists on changing eligibility criteria under successive policies (Castles 1994, Productivity Commission 2002, Marston and McDonald 2007, Considine *et al*. 2014, Department of Employment and Workplace Relations 2024). Less is known about the social, health, and equity impacts across the five policy iterations; a gap in the literature that this paper seeks to address. The aim of this paper is to incorporate findings across these iterations to answer the research question:

What are the social, health and equity impacts from the privatization of employment services in Australia?

## Materials and methods

Our study was based on document analysis which allowed us to assess the ways in which the privatization of the CES from 1994 has credibly affected health and equity. Document analysis is a well-established qualitative research method involving the systematic examination of documents which can include policy briefs, contracts, government reports, programme guidelines, and evaluation studies—to uncover context, discourse, decision-making processes, and underlying assumptions (Bowen 2009). Bowen notes that ‘Document analysis yields data, excerpts, quotations, or entire passages, that are then organised into major themes, categories, and case examples specifically through content analysis’ (2009, p. 28). The document analysis provided us with access to rich, contextually bound data which grant retrospective insight into policy design, implementation, and rationale that are less accessible through interviews or observations alone (Kayesa and Shung-King 2021).

Morgan (2022) notes that document analysis is underused in qualitative research, even though it can allow a powerful lens to understand policy historical evolution, actors’ framing, and embedded values. Given that we were researching policy change over a 30-year period, interviews with policy actors would have been difficult and very heavily subjected to interview recall. In contrast, a document analysis allowed a means to survey changes over 30 years, and through critical interpretive policy analysis (van Hulst *et al*. 2025) determine the pathways from the privatization of the services to their health and well-being impacts.

A critical interpretative research approach also enables us to identify issues of subjugation and power and links people's experiences of unemployment to broader political, economic, and cultural systems (Jacobs 2021). A critical approach is appropriate for undertaking research which supports aspirations for social change and for improving public health.

### Data collection

We were informed in our selection of data by the types of sources which have provided key insights on the health impacts from privatization and outsourcing of government roles to the private sector more broadly. These include indicative government reports, submissions to government inquiries, civil society surveys, academic literature, and credible online media and policy platforms. This approach was adopted as an empirical study of people’s experiences and health and equity outcomes has not been conducted. It would have been methodologically very difficult to complete because of the near impossibility of controlling for a range of variables likely to impact health. Consequently, we have drawn on a range of different documentary sources which enable us to study health impacts and draw conclusions about the impact of the process of privatization (Bowen 2009). Data sources and their selection are listed in Table 1.

**Table 1:** Data sources and selection.

Data sources and selection
1. Government reports Productivity Commission (2002): Independent Review of the Job Network Senate Inquiry (2019): Education and Employment References Committee: ‘Jobactive—failing those it is intended to serve’ Parliament of Australia (2023): ‘Rebuilding Employment Services’: Final report on Workforce Australia Employment Services—House of Representatives Select Committee on Workforce Australia Employment Services These three final reports were selected to provide insights into documented social and health impacts across different iterations of a privatized policy spanning 20 years. This was to highlight any important similarities and differences across the documents and to identify important context.
2. Selected submissions to inquiries into Job Active and Workforce Australia All submissions to the inquiry into Job Active and Workforce Australia (above) were examined with the aim of augmenting the reports’ more general findings. Selected submissions chosen to prioritize the voices of unemployed people, including those who had sought anonymity due to fear of speaking out (*n* = 11). The views of civil society actors, Non Government Organisations (*n* = 7), academics (*n* = 2), policy organizations (*n* = 3), and a union representative (*n* = 1) were all prioritized for inclusion as they specifically highlighted social, health, and equity impacts of privatized employment services.
3. Civil society surveys of unemployed people Australian Council of Social Services (2022): ‘Voices 2: results of a survey of people who used jobactive 2022’ Antipoverty Centre (Coonan and O’Connell 2023): ‘Punishment for profit: How private providers became the only winners in Australia’s cruel employment services system’ These two recent surveys from key civil society actors were specifically selected as they provide rich data from voices of unemployed people concerning enduring social and health impacts of privatization of employment services. They also provided indicative nuanced critical analyses of privatized employment services.
4. Academic literature Selected academic literature and reports were included from the ‘Australian Welfare and Work Lab’ at the University of Melbourne where academics have built a programme of policy-engaged research and monitoring the impacts of welfare reform on the frontline delivery of employment services in Australia and internationally for over 20 years. Academic literature was specifically limited to this cohort of researchers as the leading research reporting impacts of privatized service provision from the perspectives of both unemployed people and service providers.
5. Online media and policy platforms Media and policy sites offering critical analyses of privatization of employment services and welfare-to-work policy were examined, using key search terms including ‘employment services and ‘privatized employment services’. These broad search terms were selected to capture all appropriate items discussing social, health, or equity issues associated with employment service provision. No date restrictions were applied to searches to allow for the full reporting timeframe. Items identified included expert commentary from academics, investigative journalists, policy experts, campaigners, and advocates who were reported in the following online platforms: Michael West Media (investigative journalism) The Guardian (Australian edition) The Saturday Paper The Conversation Pearls and Irritations Independent Australia These platforms report on a wide range of social, economic, and policy issues, including employment and privatization

### Data analysis

Ninety-two items were imported into Nvivo qualitative data analysis software (version 14) and analysed thematically using an interpretive policy analysis approach (Braun and Clarke 2006). Our analysis involved gaining familiarization by reading the data, collectively identifying an a-priori coding frame (Supplementary Material S1), coding each of the 92 documents, then mapping and collaboratively interpreting the data (Braun and Clarke 2006). The rapidly changing policy iterations and the limited related social and health outcome data preclude attributing the impacts to any one specific iteration. Instead, we sought to focus on the overarching social, health, and equity impacts, over time. This research is thus generic in providing a broader understanding of these impacts over 30 years.

Team meetings were convened to analyse the coding frame and identify key themes. The criteria for theme inclusion were adopted reflexively and included both salience and relevance for answering the research question. It was important to understand the historical, political, and ideological contexts impacting service provision and how this affects social, health, and equity outcomes. We were aware of our positionality as social and commercial determinants of health and health equity researchers committed to social justice and sought to embrace a reflexive approach to the analysis through team meeting discussions to review our interpretations, and explore alternative viewpoints and explanations (Braun and Clarke 2019, 2023). We acknowledge that as researchers, our theme identification and analysis cannot be completely divorced from our positionality which also informs our critical research perspective (Holmes 2020).

## Results

Table 2 presents initial results that were collated to contextualize the broader research findings. It outlines the changing landscape of employment services between 1946 and 2023, including the timeline of successive governments, their key policy features, and rationales for adoption.

**Table 2:** The changing landscape of employment services in Australia 1946–2023.

Policy	Government and timeline	Key features	Rationale for adoption
Commonwealth Employment Service	Curtin Labor Government 1946–94	Acted as a centralized labour exchange. It informed unemployed people of employment vacancies nationally and allowed employers to survey all available labour. It also helped employed people to find better work.	To achieve full employment. The 1945 White Paper on Full Employment identified the need for a publicly managed, supportive employment service and labour exchange to deal with the extent of post-World War II unemployment.
Working Nation	1994–98 Keating Labor Government	Introduced private employment providers with two-thirds of unemployed people moving from the public service to a mix of private and not-for-profit providers. Case management and training programmes were provided for those in long-term unemployment, underpinned by a Job Compact.	A growing focus on competition and a less rigid programme, with individual case management. ‘Reciprocal obligations’ were imposed to engage in education, training, or other forms of productive work. The government’s reciprocal obligations were to provide the supports needed to facilitate employment.
Job Network	1998–2009 Howard Coalition Government	Introduced full competition in employment services, with greater emphasis on paying private service providers for achieving employment outcomes. Remaining public agencies were closed, and Centrelink introduced as an arm-length agency to manage social security payments. Cuts to training programmes and other services provided under Working Nation, with enforcement of ‘mutual obligation’ to encourage active workforce participation. ‘Work for the dole’ introduced for those aged 18–24 years. Three hundred providers delivered Job Network contracts between 1998 and 2009.	The rationale was to prioritize a strong ‘outcomes focus’ for service delivery to address structural weaknesses and inefficiencies. Greater competition was deemed to drive greater efficiency for the taxpayer and increased choice for jobseekers.
Job Services Australia	2009–2015 Rudd-Gillard Labor Government	A shift from a sole focus on ‘Work for the dole’ to other forms of mutual obligations including volunteering and training. One hundred and forty-one providers delivered Jobactive contracts between 2009 and 2015.	A shift in emphasis from a punitive model and to replace Job Network’s ‘one size fits all’ approach and allow for greater flexibility for employment services providers.
Jobactive	2015–22 Abbott Coalition Government	Return of ‘Work for the Dole’ for those under 50 years. Introduction of a quasi-market with purchasing power centralized in a single government agency. Forty-four providers delivered Jobactive contracts from 2015 to 2022 from 1700 sites.	A return to imposing stronger obligations reflecting a shift from an ethic of ‘entitlement’ to one of ‘responsibility’.
Workforce Australia	2022: ongoing Morrison Coalition Government	Support for eligible individuals to access online tools and resources based on their own personal needs. Individuals who require additional support receive tailored support. Assistance is provided for businesses to find suitably skilled staff. Forty-three providers have delivered Workforce Australia services since July 2022 from 1482 site locations.	To place significant emphasis on moving digitally literate job seekers away from using private service providers towards a web-based self-service obligations scheme. A subsequent 2023 review of Workforce Australia by the Albanese Labor Government found that decades-long full privatization of employment services has failed and proposed a future role for a public provider. This will not be the recreation of the CES but a new entity, Employment Services Australia, with an enduring physical presence in local communities.

Sources: Productivity Commission 2002, O’Sullivan *et al*. (2021b), National Employment Services Association n.d., Workforce Australia employment services—Department of Employment and Workplace Relations, Australian Government online.

Our document analysis identified five key interconnected themes which address the research question: (1) ideological underpinnings, (2) the primacy of private interests, (3) impacts of privatization on service quality, (4) negative social and health impacts, and (5) implications for equity.

### Theme 1: ideological underpinnings

Ideological standpoints underpin privatization of employment services, with the logic of NPM mainly concerned with adopting private sector techniques of budgeting and planning in the 1970s and 1980s. By the late 1980s and early 1990s, Australia used contracting out and privatizing to shift the administrative burden to nonprofit and for-profit sectors (Considine and Lewis 2010). Beginning under Job Services Australia, unemployed people aged between 18 and 29 years were also required to undertake ‘Work for the Dole’. This is a work experience programme that has been widely associated with the derogatory term ‘dole-bludger’ (Anaf *et al*. 2024), Australian slang for a person who collects unemployment benefits but who is perceived as making no serious effort to find work.

Proponents of privatized policies argue that work experience programmes lead to service innovation, cheaper services, and greater service quality and responsiveness (Osborne and Gaebler 1992). In other countries including the UK and the USA, welfare benefits were ideologically framed as potential poverty traps’ that keep people dependent on state provision (Le Grand and Bartlett 1993). Therefore, employment policies provide limited cash resources, both in value and duration, and are based on strict conditionality (Bonvin 2008).

One mechanism deemed to achieve greater competition and efficiency is increasing the diversity of providers by facilitating delivery of public services through the private sector or by creating quasi-markets (Le Grand and Bartlett 1993). However, as Marston and MacDonald (2008) contend, clients engage with private agencies as a formal requirement, not necessarily because they believe that they will have their needs met. Arguably, privatized agencies and their clients belong to a structure of domination and systemic ‘carelessness’, which is largely (using Bourdieu’s theory) ‘misrecognized’ as caring (Peillon 1998, Bowman *et al*. 2016). They are underpinned by an emphasis on ‘activation’, the policy concept which emerged in response to criticisms of welfare states in the 1960s and 1970s, promoting the primacy of work.

Benefits are linked to ‘active’ participation in either unpaid work or other activities that will improve the likelihood of gaining employment (Andersen and Larsen 2024). Unemployment benefits are deemed to reduce workers’ incentives to take jobs, and incur costs that reduce capitalists’ incentives to invest (Greer 2016 ). Activation casts unemployed people as inherently passive, with a normative claim that activation benefits the recipient (Caswell *et al*. 2024).

NPM accountability instruments impose a form of ‘double activation’ affecting both unemployed people and frontline workers; an interplay which is both a critical and distinctive form of contemporary poverty governance (O’Sullivan *et al*. 2019). Activating the unemployed becomes a depoliticized issue of individual attributes rather than one of available jobs and the role of public policy (Marston and McDonald 2008). Activation assumes that individual purposeful action will suffice to overcome unemployment. However, this demands that unemployed people face coercive financial sanctions to ensure their compliance, and that they also self-identify as ‘job seekers’ rather than as being currently unemployed, with the associated emotional work to adopt this identification (Hochschild 1983). As research of longer-term unemployed people by Peterie *et al.* (2019) found:

As activation’s feeling and framing rules would predict, emotion work was typically performed with one of two objectives in mind. First, interviewees sought to manage debilitating feelings of shame and failure, so that they would not fall into despair and compromise their job search efforts. Second, interviewees tried to mask or diffuse socially unacceptable feelings of anger, which may again be understood as corrosive to their employment prospects.

Transforming citizens from ‘passive’ welfare recipients into ‘active’ labour market participants or ‘job seekers’ signifies the basis of the institutional character of many twenty-first century welfare states (Marston and McDonald 2008).

### Theme 2: the primacy of private interests

There are many ways in which marketized services affect the social determinants of health, by promoting private ahead of public interests and undermining the belief that the role of governments is primarily to serve the broader population (Johnston 2019). However, more than Au$9.5 billion in taxpayer funds will be spent over the next few years on private employment service providers (Kelly 2024b), with employment services being the largest single government procurement after Defence (Parliament of Australia 2023). Privatized employment services are becoming increasingly involved in wide-ranging fields of human service provision, including disability, healthcare, training, housing, and food banks (Antipoverty Centre Inc 2023). This has:

… created a feedback loop whereby it seems that providers are now deemed too big to fail by government. They have influence over the policies that guarantee their income streams without meaningful pushback from the politicians who legislate the programs (Antipoverty Centre Inc 2023).

This is also indicative of the extent to which these entities have increasingly become powerful commercial determinants of health (Gilmore *et al*. 2023).

Job Services Australia (2009–15) which superseded Job Network (1998–2009) was a Au$1.3 billion scheme (Besser *et al*. 2015). A media investigation found that the Federal Government had recovered >$41 million in false claims over 3 years by private employment agencies due to strategies including ‘rorting’, manipulation of records, forgery, and inflated claims (Besser *et al*. 2015). Under the next policy iteration, Jobactive (2015–22), the system had become increasingly dominated by large multinational corporations (Tull 2023). Three of these together accounted for 74% of the $3.3 billion in government tenders awarded under that model (Morton 2020, Foote and Tran 2021b). AusTender reports show that the largest private providers were Max Solutions and Advanced Personnel Management (APM), both of which are owned by separate, American-based multinational investment firms. These entities received over $2 billion, or 60% of all Jobactive contracts (Foote and Tran 2021b), with the profits of this financialization of the service flowing to the investment firms’ shareholders (Friel *et al*. 2024).

There are good reasons to promote the private sector, which is a source of economic growth, jobs, and incomes. A positive policy response to the COVID-19 pandemic was therefore government funding through the wage subsidy programme, JobKeeper, which assisted businesses to cover employees’ wages and allow for their retention under ‘lockdown’ conditions. However, Jobactive providers were also paid bonuses for placing people who theoretically remained employed after the $87 billion JobKeeper payment was announced. This allowed a perverse incentive for private agencies to ‘double dip’, resulting in a substantial impost for taxpayers and a ‘potential goldmine for the private agencies’ (Morton 2020).

Our research also identified a range of other strategies used by such firms, including political donations. The Australian Electoral Commission Transparency Register shows that APM was a key political donor during the 2019 federal election, contributing $119 673 to the Liberal (conservative) Party and $22 372 to the Labor Party (Foote and Tran 2021b). The Australian-based Sarina Russo Group which received almost $80 million in government contracts, donated $60 500 to the Liberal and National Party coalition Government (Foote 2022).

Profitability relies on obtaining fees that stem from participants’ mutual obligation requirements, including undertaking Employability Skills Training, Career Transition Assistance, or other chargeable activities (Per Capita 2023). Concern with profitability encourages providers to direct ‘jobseekers’ towards options that are not necessarily in their best interests. Some job agencies hold as many as 12 related entities to which they can refer including recruitment firms, registered training organizations, psychologists (Morton 2023), human resources firms, and career transition services.

Our findings also unearthed strategies fostered by a range of incentives that highlight conflicts of interest in private provision, including skills training sessions that are run by the private agencies but paid for from the government’s Employment Assistance Fund (Tull 2023, Per Capita 2023, Australian Government, Dept Social Services 2024). However, this fund is designed to provide unemployed people with practical assistance for job-searching activities. Yet agencies can profit from these funds by forcing clients who are on a Job Plan to attend (Brennan 2020), even if sessions are generic and unhelpful (Australian Youth Affairs Commission 2023).

A further strategy identified in the inquiry into Jobactive (Parliament of Australia 2019a) was service providers claiming outcome payments for employment placements made without their assistance (Molloy 2019). Tactics included client inducement and harassment to secure copies of payslips which would provide evidence in support of receiving commissions (Parliament of Australia 2019a). As one respondent argued in a media investigation into Jobactive, and reported by Molloy (2019):

I told them I found work, and they started hassling me for pay slips and my boss’s contact details so they could get their commission. Why should they get taxpayer money when they did absolutely nothing? For months they treated me like s**t … I genuinely wanted work. They did nothing but they wanted money.

This reveals the competing interests and perverse incentives under the enduring policy logic underpinning the devolution of government services (Lipsky 1980, van Berkel 2014, Kelly 2025), including a decrease in successful placement rates (Kelly 2025).

### Theme 3: impacts of privatization on service quality

Our analysis noted how the different policy iterations have been beset by ‘system failures, perverse outcomes, unfair rules and rule-breaking by providers’ (Coonan and O'Connell 2023, p. 20). These affect service quality by impacting employment service users, service providers, their workers, and employers.

#### Employment services users

Perverse incentives negatively affect unemployed people. Strategies that impact service quality include risk selection, which includes ‘creaming’ (profiting from those who require little assistance, or the ‘cream’) and ‘parking’ (giving less assistance to more expensive high needs interventions; Parliament of Australia 2019a, Brennan 2020, Foote and Tran 2021b, Department of Employment and Workplace Relations 2023). Risk selection has also been reported in the UK where performance-based contracts led to ‘parking’ (Koning and Heinrich 2013).

Much literature suggests that for street-level bureaucrats functioning in traditional public administration contexts, risk selection is a normal strategy of workers who are coping with large caseloads and scarce resources. This includes the experience from The Netherlands which was one of the first countries to outsource employment services to for-profit organizations (Lipsky 1980, van Berkel 2014). Implications for welfare recipients therefore ultimately rest on the public values embodied in programme goals, regardless of whether preferences aim to ensure equity of access to publicly funded services or instead increase efficiency and maximize returns to taxpayer investments (Koning and Heinrich 2013). As private providers are ‘shielded from public scrutiny by contractual commercial-in-confidence provisions, this also protects them from freedom of information requests’ (Rexter 2018) which support more transparent and accountable government. The level of service quality ultimately reflects a range of transaction costs which:

…disturb established trusting relations with jobseekers and employers as providers enter and exit the market. This corrodes or destroys existing relationships and networks for jobseekers, employers and communities (Brotherhood of St Laurence *et al*. 2023, p. 16).

The impact of perverse incentives and lack of public scrutiny is particularly concerning given the high level of payment suspensions imposed on service users. In the 2022–23 financial year alone, 1 750 923 Jobseeker payment suspensions were imposed for an undisclosed number of clients, mainly due to failure to meet mutual obligation requirements (Kelly 2023). However, two decades earlier, the Productivity Commission in its evaluation of Job Network (2002) had noted the deleterious impacts of such penalties. These also disempower unemployed people who do not have motivational difficulties but might be compelled to comply with unhelpful directions or unreasonable requests.

#### Service providers and their workers

We found that service providers are also impacted negatively (Eardley 2003, Parliament of Australia 2019a, O’Sullivan *et al*. 2019, Parliament of Australia 2023). Frontline human service providers are part of a ‘street-level’ bureaucracy (Lipsky 1980) holding specific roles and responsibilities with implications for the agency, health and well-being of both welfare recipients, providers themselves, and ultimately the broader community. They are often women living in vulnerable circumstances (O'Sullivan *et al*. 2021b).

Gender can be a significant determinant of health experiences and outcomes (Department of the Prime Minister and Cabinet n.d.). Under current Workforce Australia policy, 78% of caseworkers are female, and 97% are nonunionized (Ball *et al*. 2023). The gendered nature of their work requires engaging in the emotional labour (Hochschild 1983) that is required to motivate, coax and discipline their clients (O'Sullivan *et al*. 2021b).

As Marston and MacDonald (2008) report, there is a sense of real pressure for both employment consultants and the long-term unemployed, as providers must maintain their viability under a marketized service model. Although there are some excellent frontline workers, they ‘rarely last long in the system’ (O’Halloran *et al*. 2021, p. 605). The Productivity Commission (2002, p. 17) had earlier noted the ‘depersonalising behaviour and emotional exhaustion of case managers’ under Job Network. Their report doubted whether unemployed people receive a better quality of support than they had under the superseded CES. Two decades later, researchers noted how the profiles of frontline workers have changed, as they had:

… encountered a system staffed by people with little specialist skills and job security. When job services were privatised 30 years ago, many frontline staff came from a professional or social work background. Today… case workers are former hairdressers, bakers, flight attendants, hospitality workers and carpenters. Some have been long-term unemployed themselves. The pay is low…and the turnover is high. This inevitably means those who really need help are not necessarily receiving a specialist service (O'Sullivan *et al*. 2021a).

#### Employers

Our research found that employers also experienced poor service quality even under the latest policy iteration of Workforce Australia (2022 onwards) as:

Employers have fled the system, dodging floods of inappropriate job applications. Providers are forced by the payment and performance frameworks to repeatedly try to place jobseekers into unsuitable vacancies to chase outcomes payments so they can pay their staff and make a profit, yet there are inadequate incentives or support for business to take on disadvantaged jobseekers (Parliament of Australia 2023, xii).

The review into Workforce Australia (Parliament of Australia 2023) noted that marketization led to low service quality for employers, as onerous job search requirements lead to numerous low-quality employment applications which deplete employers’ resources and increase negative perceptions of jobseekers. A parliamentary submission into Workforce Australia by Per Capita (2023) explains that by November 2022 employers had directly registered only 3.2% of vacancies from a total of 200 000 on the new Workforce Australia online platform which had been designed to simplify employers’ vacancy listings.

### Theme 4: negative social and health impacts

Our research enabled us to document the direct social and health impacts which were identified from personal testimonies, research, media reporting, reports by civil society actors, and government publications (Marston and McDonald 2008, Parliament of Australia 2019b, Australian Council of Social Service 2021a, Henriques Gomes 2022, Anonymous 2023, Per Capita 2023). They include reports of emotional ‘scarring’, loss of self-esteem, self-harming and suicidal ideation, stigma and shame, and lack of perceived control.

One negative mental health impact that especially affects the long-term unemployed is ‘scarring’, whereby the longer people remain unemployed their chances for finding work recede. This leads to a loss of social connectedness (Casey and Lewis 2020). The Economic Inclusion Advisory Committee (2024) states that mutual obligation programmes also lead to stress, loss of confidence, and thus employability.

Self-esteem is the positive self-regard that promotes a sense of approval and success and acts as a spur to agency (Bandura A, National Institute of Mental Health 1986). This can be negatively affected by the prevailing policy environment, as one unemployed person maintained in their submission to the parliamentary inquiry into Jobactive:

The thing that holds me back more than anything is my lack of self-esteem. My confidence is completely shattered…I have mental health issues that I cannot afford to get treatment for, even with the rebate… I feel stressed constantly. I feel isolated, judged and incredibly alone (Anonymous n.d.).

We also found reports of self-harming and suicidal ideation under both Jobactive (Anonymous 2023, Parliament of Australia 2019b) and Workforce Australia (Coonan and O'Connell 2023) employment service models. As one anonymous person stated:

I was physically and emotionally exhausted, and I couldn’t see any way out of my personal problems… I tried to take my own life. It was after this attempt, that I saw a new GP…But the damage was already done. I have absolutely no faith or respect for the Jobactive system. I have continually been put down, embarrassed, humiliated and made to feel like a failure by system.

As submissions to the Workforce Australia inquiry argued, employment services are neither trauma-informed (The National Council for Single Mothers and Their Children Inc 2022), nor do they cater for the particular needs of young people (Australian Youth Affairs Commission 2023). The now discontinued ParentsNext policy, which imposed mutual obligations on parents of young children, had disproportionate negative social and mental health impacts on Aboriginal and Torres Strait Islander women who were experiencing domestic violence (Australian Council of Social Service 2021). Programmes which instead provide generous and long-lasting benefits, such as in Switzerland and Scandinavian countries enable capacity for personal action towards securing employment (Sen 1999, Bonvin 2008).

Actions from job agencies resulting in bullying and harassment, humiliation, stigma and shame, stress, and lack of perceived control were also cited by unemployed people as affecting their health (World Health Organisation 2008). An anonymous submission to the inquiry into Jobactive reported that the main reason for choosing to come off unemployment benefit payments was to avoid dealing with service providers:

I still feel furious with them for the stress they caused me by the punitive, ill-equipped and aggressive way that they dealt with me.

Another person noted both social and health impacts, whereby:

The stress from being cut off and having threatening appointments eventually led to me having panic attacks. I was terrified of being watched. I could imagine my every-day life being construed as non-compliance and my payments being suspended at random. I had internalised the compliance nature of the mutual obligations and it aggravated my depression. My life was spiralling out of control.

We also found reports of negative social and health impacts for service providers, with one unemployed person stating:

My own case manager has 200 people on his caseload. His job is to observe welfare compliance, issue penalties, and manage the paperwork for the Government kickbacks. Most case managers I have spoken to are working in the welfare industry because they couldn’t find any other work that paid them enough to live on. Many are barely hanging on to their own jobs. Having retail and sales as their only prior work experience, their current work environment is highly stressful (Anonymous 2023).

### Theme 5: implications for equity

Equity in health is an ethical value which is inherently normative, grounded in the ethical principle of distributive justice, and aligned with human rights principles (Braveman and Gruskin 2003). Global evidence shows that the lower an individual’s socioeconomic position, the worse their health is, resulting in a health gradient (World Health Organisation 2024). This describes the phenomenon whereby people who are less advantaged in terms of socioeconomic position have worse health (and shorter lives) than those who are more advantaged (World Health Organisation 2008). This is a global phenomenon, observed in low, middle, and high-income countries (World Health Organisation 2008).

Although the health gradient means that health inequities affect everyone, as the Inquiry into Workforce Australia (2023) acknowledged, certain cohorts are disproportionately negatively impacted by barriers to social and economic participation. People from culturally and linguistically diverse (CALD) backgrounds, those who are migrants and refugees, those with caring responsibilities (mainly women), those who live with disabilities, and those of mature age all face inequitable employment barriers (Australian Council of Social Service 2021a, Parliament of Australia 2023).

Aboriginal and Torres Strait Islander peoples are also differentially impacted, experiencing four times the proportion of unemployment than the wider population. Lower levels of digital inclusion also exacerbate difficulties in accessing employment services (National Indigenous Australians Agency 2023), as does lower access to education and training opportunities which are compounded by high incarceration rates (Parliament of Australia 2023).

Despite differential pricing structures that apply to providers upon meeting a range of outcome triggers and incentives in a marketized employment system, Australia has arguably failed historically to deliver good results for unemployed people with complex needs. The average duration for engagement with employment services for people who are highly disadvantaged is ∼5 years (O’Sullivan *et al*. 2019). Therefore, whatever coercion and obligations are imposed, many unemployed people will remain financially ‘dependent’ on the state. This is because of adverse social determinants of health, including:

Homelessness, illiteracy, and chronic health problems cannot be wished away by pronouncements about the need for greater reliance. In light of what people have told us about the experience of workfare programs, the self-efficacy argument for increased compulsion starts to sound very hollow (Marston and McDonald 2008, p. 266).

Thus, such equity groups will unfairly experience the negative health impacts described above under enduring privatized service provision.

## Discussion

While primarily focused on Australia, this work has international significance and produces evidence relevant to the work of organizations like the International Labour Organisation (ILO) and its Decent Work agenda (International Labour Organisation 1999, 2023). This agenda highlights the importance of employment services for overcoming labour market inequality. Although a ‘work first’ approach may impact favourably on employment statistics and improve employability through work experience, it risks both job quality and sustainability for service users (International Labour Organisation 2023). A common objective of labour market policies is to enable people to access better quality jobs with higher wages, and also offer better career prospects and thus help to improve equity (International Labour Organisation 2023).

Our research also highlights both the importance of governance arrangements and the interlinkages between different strategies of the Ottawa Charter for Health Promotion, especially the need to build healthy public policy and create supportive environments to ensure adequate employment services (World Health Organisation 1986). It provides a critical analysis of social, health, and equity impacts arising under the marketized employment services model adopted after disbandment of the CES in Australia, and the retreat of government from direct employment service provision in many global jurisdictions including the UK, USA, France, and The Netherlands.

Participants in Australia’s own quasi-market are the Federal Government as the ultimate purchaser of services, the Centrelink agency as the services allocator, the privatized agencies which sell their services, and unemployed people who are the service users. There endures, therefore, an inherent tension between service price and quality (Buragess 2003) spanning different policy iterations. Although negative social, health, and equity impacts were evident throughout the 30 years of privatized employment services, we did not find such impacts from the CES in our data. However, as research in Chicago reported by Brodkin (1997) highlighted, under welfare delivery devolved to the state level, clients also had little capacity to hold the state accountable for providing specified levels of service quality. In a neoliberal political and policy environment which is increasingly opposed to social support, and with pressure to ‘meet the numbers’ under inadequate budgets, the purported ‘welfare contract’ between the recipient and the state is ‘virtually unenforceable’ (Brodkin 1997, p. 20).

Negative impacts identified in our research also include the loss of public funds which are sent offshore by multinational service providers which could instead be used for social and health investment in Australia (Tran 2021). The lack of trained staff, combined with a demanding role, results in high staff turnover and compromised quality of support for unemployed people (O'Sullivan *et al*. 2021a). A marketized model has inherent conflicts of interest and perverse incentives that result in poor agency practices and client outcomes (O'Sullivan *et al*. 2021b). Our research revealed conflicts of interest including the practice of service providers claiming payment outcomes for placements that they did not facilitate (Kelly 2024a).

Negative impacts partly reflect how growing financialization has altered the way that governments provide public services (Thomson and Dutta 2018). Financialization introduces uncertainty in the commitment to public service provision (Epstein 2005, Friel *et al*. 2024), as multinational corporate providers focus more on shareholder and senior executive profit, to the detriment of wages and worker protection (Friel *et al*. 2024).

With a growing policy focus on easier to place unemployed people being directed to self-managing their employment transition through a digital services model and an online ‘app’, multinational service providers now expand their focus to identifying lucrative funding options available to deal with harder to place clients, including those in the disability sector (Foote and Tran 2021a, West and Foote 2021, Advanced Personnel Management 2024). However, as Loosemore (2021) contends, technology is unlikely to be effective without addressing a major flaw in the government’s approach which is premised on private competition between providers and not on collaboration. What is needed is a system of real engagement and collaboration between all parties, but many employers concerned with profit margins are disinclined to risk hiring disadvantaged job seekers through employment programmes (Loosemore 2021).

As Coonan and O’Connell argue (2023), it is important to adopt an income-first, rather than work-first approach to employment policy if unemployed people’s well-being is to be the prime consideration. A work-first approach emphasizes securing immediate employment as the primary goal, often focusing on short-term training together with stringent conditionality (Etherington and Daguerre 2015). An income-first welfare approach instead prioritizes direct financial assistance to ensure meeting basic needs, and social determinants of health such as food, housing, and healthcare, without requiring engagement in work participation or training programmes (Coonan and O'Connell 2023). This approach would help to remove poverty as a barrier to gaining paid work, by providing financial support which augments personal capacity, and also valuing other forms of social and economic contribution (Department of the Prime Minister and Cabinet 2023).

To better support health and equity, employment service governance should be informed by a capabilities approach (Sen 1999), acknowledging the two necessary preconditions for responsible behaviour in gaining employment. These are having sufficient means and power to act, and relative freedom to choose one’s way of living (Sen 1999). This approach would allow for developing genuinely effective and supportive employment services (Bonvin 2008, Coonan and O'Connell 2023).

As our historical overview shows, over time privatization of employment services has failed to improve the prospects of Australians seeking work and has damaged their health. The Economic Inclusion Advisory Committee (2024, p. 69) argues, ‘that era has outlived whatever usefulness it may have had and has become an economic, social and human burden we just can’t afford’. Echoing this, the inquiry into Workforce Australia gave 75 recommendations for designing a new employment services model including a much stronger government role (Parliament of Australia 2023).

The ILO also argues that governments can generate employment by expanding social services and by investing in social determinants of health through health, education, and other sectors. This is not only to create immediate jobs but also to realize the long-term gains of a healthier and more highly skilled and educated population, with implications for better standards of living and productivity, thus stressing the importance to health promotion more generally (International Labour Organisation 1999).

A growing case is being made internationally for remunicipalization (Public Services International 2024) or renationalization (Quiggin 2017) to return selected privatized services to public ownership and control. This would allow them to be managed in the public interest, not for corporate profit. It is time for a similar conversation in Australia.

### Research strengths and limitations

The strength of this research is providing an integrated historical overview of the social, health and equity impacts from privatized employment policy spanning 30 years in Australia from a social determinants of health perspective. It highlights the conjunction of the power of neoliberal ideology promoting privatization of employment service provision and increased welfare conditionality which undermines human agency. While focusing on the Australian context it is informed by insights from other global jurisdictions. No data are available that would enable a study of the health impacts on users of the services directly and the document analysis uses accepted methods to study the impacts of policy implementation retrospectively.

## Conclusion

To support the ILO’s call for decent work for all and to support health promotion, governments should address those negative aspects of private service provision that lead to negative health, social, and equity impacts, including poverty and severe emotional distress which unfairly affect people in disadvantaged circumstances. In the 30 years, since privatization of employment policy, changing iterations have failed to implement necessary reforms to address systemic failures. This particularly impacts unemployed people and also the frontline service providers who support them under system and financial constraints, as well as employers. It is time to heed the call for a much greater direct government role in employment policy, increased regulation of private providers, or even the return of employment services to public ownership and control as a prerequisite for supporting health and equity, and public over private interests.

## Supplementary Material

daag005_Supplementary_Data

## Data Availability

The data underlying this article will be shared on reasonable request with the corresponding author.
