# Supplementary material for: Privatized employment services in Australia: addressing social, health, and equity impacts for health promotion
Source: Health Promot Int. 2026 Feb 2;41(1):daag005. doi: 10.1093/heapro/daag005 (PMC12862640; doi:10.1093/heapro/daag005)
Supplement: daag005_Supplementary_Data [file daag005_supplementary_data.docx]

**Coding frame**

**Social and health impacts from privatisation of employment services in Australia**

To support the thematic analysis for a critical understanding of the impacts of privatisation of employment services in Australia

| **Nodes** | **Context** |
| --- | --- |
| Challenges faced by job-seekers | References to challenges faced by job seekers in accessing employment services under a privatised model. This includes job seekers’ responses to any difficulties faced in compliance with mutual obligations requirements. |
| Challenges faced by frontline service providers | References to challenges faced by frontline service providers in delivering employment services under a privatised model. This includes ways in which the system is not fit-for-purpose. |
| Benefits of privatised employment services | References to any positive aspects of privatised employment services |
| Strategies employed by job agencies | Strategies to facilitate the financial interests of private job agencies (eg ‘creaming’ and ‘parking’). This includes any perverse incentives to facilitate profit-seeking |
| Conflicts of interest | Conflicts arising for agencies and frontline service providers |
| Lack of accountability | References to private job agencies evading government sanctions for poor performance or other negative outcomes. |
| Lack of transparency | References to lack of transparency by private job agencies and /or frontline workers and by government |
| Fraud, corruption | References to fraudulent or corrupt activities by privatised job agencies |
| Cost of privatised employment services | References indicating the cost disparity between government and private provision of employment services |
| Health impacts for job-seekers | References to health impacts arising from privatisation of employment services. This includes distress and other mental health impacts. |
| Health impacts for service providers | References to health impacts arising from privatisation of employment services |
| Implications for equity | References on implications for equity from privatised employment services |
| Private vs public interests | References on insights on prioritising private over public interests |
| Managerialism / neoliberalism | References aligning the privatised services with New Public Management or neoliberal ideology |
